# Metal Oxide Gas Sensors to Study Acetone Detection Considering Their Potential in the Diagnosis of Diabetes: A Review

**DOI:** 10.3390/molecules28031150

**Published:** 2023-01-24

**Authors:** Yasser H. Ochoa-Muñoz, Ruby Mejía de Gutiérrez, Jorge E. Rodríguez-Páez

**Affiliations:** 1 Composites Materials Group (GMC-CENM), Universidad del Valle, Cali 76001, Colombia; 2 CYTEMAC Group, Universidad del Cauca, Popayán 190003, Colombia

**Keywords:** acetone, volatile organic compounds (VOCs), gas sensors, metal oxide (MOx), micro/nanostructures, diabetes

## Abstract

Metal oxide (MOx) gas sensors have attracted considerable attention from both scientific and practical standpoints. Due to their promising characteristics for detecting toxic gases and volatile organic compounds (VOCs) compared with conventional techniques, these devices are expected to play a key role in home and public security, environmental monitoring, chemical quality control, and medicine in the near future. VOCs (e.g., acetone) are blood-borne and found in exhaled human breath as a result of certain diseases or metabolic disorders. Their measurement is considered a promising tool for noninvasive medical diagnosis, for example in diabetic patients. The conventional method for the detection of acetone vapors as a potential biomarker is based on spectrometry. However, the development of MOx-type sensors has made them increasingly attractive from a medical point of view. The objectives of this review are to assess the state of the art of the main MOx-type sensors in the detection of acetone vapors to propose future perspectives and directions that should be carried out to implement this type of sensor in the field of medicine.

## 1. Introduction

The analysis of exhaled human breath has long been a subject of study, making use of several different methods and techniques. Since the time of Hippocrates of Cos (460 BC), physicians have known that the scent of human breath can provide clues to the diagnosis of disease. Among the identified indicators are the sweet, fruity odor of acetone in patients with uncontrolled diabetes; the musty, fishy reek of liver disease; the urine-like smell that accompanies failing kidneys; and the putrid stench of lung abscesses [1]. In 1798, John Rollo described the odor of decaying apples in exhaled breath, and 59 years later (1857), this odor was identified as acetone [2], which was then used as the first biomarker of diabetic comas. Over the years, exhaled acetone was underestimated, mainly because there were no devices suited to its detection. Linus Pauling, however, later published an article (1971) explaining the analytical methodology used to identify approximately 250 compounds in breath [3,4], and this date is, therefore, considered a starting point in the development of the analysis of exhaled breath.

**Figure 1:**
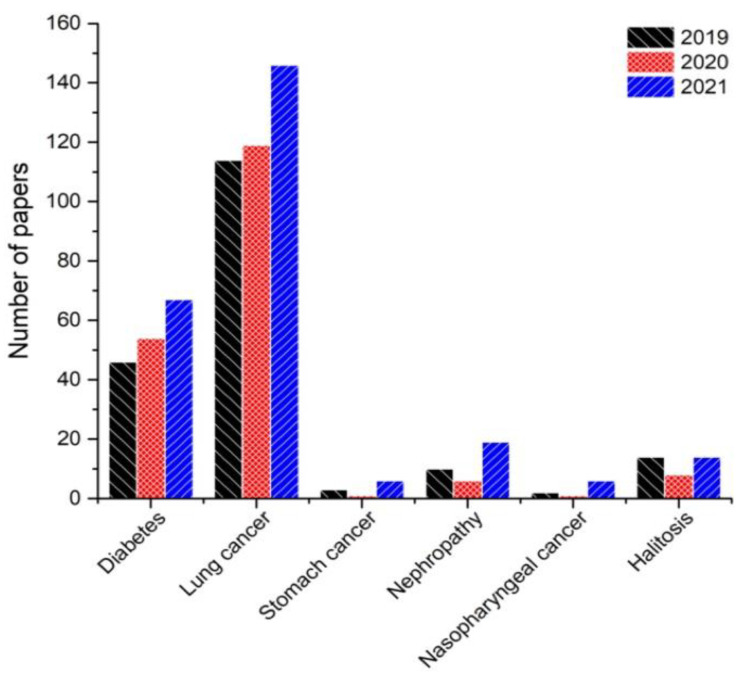
Number of papers in 2019–2021 related to the analysis of biomarkers of diseases.

In general, the methods used for the detection of biomarkers in breath are based on spectrometric methods such as gas chromatography–mass spectrometry (GC-MS) [5], proton-transfer-reaction mass spectrometry (PTR-MS) [6], and ion-mobility mass spectrometry (IMS-MS) [7]. All of the above techniques are used to measure low gas concentrations and are conducted by specially trained laboratory officers and staff. The development of nanotechnology has made it possible to consider gas sensors a potential tool for the early notification of advanced diseases [8,9]. Among these, sensors based on semiconductors metal oxides (MOxs) have advantages, such as their small size (miniaturization), low cost, easy fabrication, and good reversibility. In these devices, the electrical resistance of the gas-sensitive material is adjusted by phenomena occurring at the surface between the material, the adsorbed oxygen ions from the air, and the target gaseous compound. These devices are sensitive to a wide variety of toxic and combustible gases and have been used successfully in a wide range of fields [10,11,12]. Breath exhaled by human beings is made up of a wide variety of volatile organic compounds (VOCs) that could be used to effectively identify disorders of the body, achieving an acceptable diagnosis of disease with greater precision, as is the case of acetone vapor, which is related to diabetes [13], or compounds from the aldehydes group and ammonia vapor that are related to lung cancer and kidney disorders [14,15] and other biomarkers that have been the subject of research in recent years (Figure 1).

Traditional methods for taking blood glucose measurements during clinical examinations and home care are invasive, causing patients to experience additional psychological stress and pain [16]. In the long term, this could prove to be a disadvantage for diabetic patients, particularly those with type 1 diabetes mellitus, who are absolutely dependent on insulin injections for the treatment of diabetes. Therefore, blood glucose measurement is not a favorable method for everyone in the long run. Currently, there are no accessible, cheap, fast, hygienic, easy-to-operate, or highly sensitive methods or devices for noninvasively and painlessly detecting diabetes. The possibility of using metal oxide gas sensors in this type of application is, thus, of great interest in solving this type of problem. The presence and suitable detection of acetone gas in concentrations of 300 ppb to 1800 ppb from the exhaled breath by a person would allow for a preventive diagnosis [4]. This becomes more important when considering that the number of people with diabetes continues to increase year after year; in 2019, the number of adults with diabetes was approximately 463 million worldwide, and it is estimated that 4.2 million deaths per year are due to this disease [17]. Biomarker-based gas sensors are, therefore, the technology of choice for the early notification of advanced disease, thereby reducing the number of blood sugar measurements per day. 

There is a large number of reviews commenting on the characteristics and performance of metal oxide gas sensors for the detection of VOCs (e.g., acetone, nitric oxide, hydrogen sulfide, and ammonia). Tomić et al. [18] presented a discussion focused on key materials such as metal oxides, conductive polymers, and carbon-based materials and their mutual combination due to their representativeness in sensing VOCs. Ahmadipour et al. [19] presented breath acetone measurement techniques and the factors that affect the concentration of acetone in human breath (e.g., dietary changes, hourly variations, weight, and exercise). Yang et al. [20] and Baharuddin et al. [21], like Ahmadipour et al. [19], discussed the factors that affect detection performance, especially the structures, morphologies, presence of impurities in semiconductor materials, and specific surface area of metal oxides. Vajhadin et al. [22] and Drmosh et al. [23] presented the current state of gas sensors based on the three most common metal oxides, including ZnO, SnO_2_, and In_2_O_3_, highlighting the role of nanomaterials in improving the analytical performance of sensors. Tai et al. [24] showed the prospects for future development from individual sensors to integrated devices and self-powered health monitoring systems. The results compiled in these reviews show that the development of chemo-resistive, metal oxide-based acetone sensors is the future of noninvasive diabetes management. However, the use of metal oxides presents some challenges for chemo-resistive sensing applications, i.e., sensitivity, selectivity, limit of detection (LOD), and stability. Overcoming these challenges will lead to better diabetes control. In this review article, challenges and strategies to improve the chemo-resistive performance of a wide variety of metal oxides for the detection of acetone are presented. In addition, the need to advance the study of some materials selected from doped metal oxides and heterostructures is highlighted.

## 2. Methodology of the Literature Review

**Figure 2:**
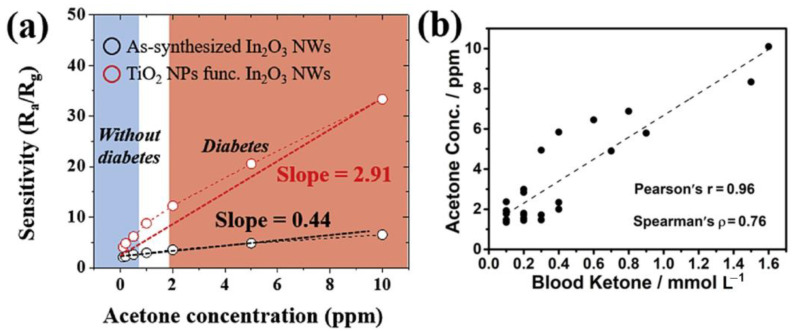
(**a**) The “healthy” and “diabetes” regions in an In_2_O_3_ nanowire sensor and a TiO_2_-nanoparticles-functionalized In_2_O_3_ nanowire sensor (adapted from [27]). (**b**) Exhaled acetone concentration vs. blood glucose concentration of diabetic patients from the Second Hospital of Jilin University (adapted from [30]).

Exhaled acetone is generally in the range of 0.3–1.8 ppm for “healthy” people and 1.25–2.5 ppm for people with diabetes [25]. Some references have shown that the level of acetone can increase to as much as 25 ppm for type 1 diabetes [26]. Figure 2a shows the response of the sensor in both regions [27]. Based on the literature review, the “healthy” and “diabetes” regions are not strictly defined, and various ranges can be considered. Moreover, in uncontrolled diabetic patients, intensified lipolysis can be found, leading to excessive ketone bodies in the blood and even ketoacidosis. When the blood ketone level exceeds 0.4 mmol/L, there is a high possibility of developing diabetes with ketosis. Therefore, the monitoring of blood ketone levels is also extremely important for diabetic patients [28]. Such control carries with it slight variation due to the complexity of the composition of human breath and the factors that influence it [19]. In recent research, the exhaled acetone concentration has been correlated to blood glucose concentration and discussed with physicians before being used as a single biomarker (Figure 2b) [29,30].

The increasing number of diabetes patients who seek to monitor their disease noninvasively—as current practice is still based on blood-sampling—has created a market for portable exhaled breath analyzers. Currently, commercially available gas sensors for acetone detection function in the order of 50–5000 ppm, values outside the range of levels of acetone exhaled by patients [31,32]. The scientific community has thus focused research on the development of more efficient gas sensors that also work at room temperature [33]. This sensor will be noninvasive and enable real-time detection. It will be less costly and miniaturized compared with standard clinical diagnostic techniques. In recent years, this area has been the subject of many publications, and, therefore, only the latest results will be shown and discussed in this review. Table 1 shows a summary of a bibliographic review in the Elsevier and Scopus databases between the years 2018–2021 of metal oxide-based sensors that have been selected for the detection of acetone considering their possible use in the diagnosis of diabetes. The keywords used for the search were “acetone gas sensor”, “metal oxide”, “VOCs”, “resistive sensors”, and “sensing performance”. The search equation for the review in the Scopus database was as follows: Your query: TITLE-ABS-KEY (detection AND acetone AND by AND metal-oxides) AND (LIMIT-TO (DOCTYPE, “ar”)) AND (LIMIT-TO (PUBYEAR, 2021) OR LIMIT-TO (PUBYEAR, 2020) OR LIMIT-TO (PUBYEAR, 2019) OR LIMIT-TO (PUBYEAR, 2018)) AND (LIMIT-TO (EXACTKEYWORD, “Acetone”) OR LIMIT-TO (EXACTKEYWORD, “Volatile Organic Compounds”) OR LIMIT-TO (EXACTKEYWORD, “Gas Sensing Properties”) OR LIMIT-TO (EXACTKEYWORD, “Metal Oxide Gas Sensors”) OR LIMIT-TO (EXACTKEYWORD, “Acetone Sensing”)). 

## 3. Description of Gas Sensors

### 3.1. Characteristics 

Numerous researchers have shown that the reversible interaction of a gas with the surface of a material is a characteristic of an active or sensitive material (MOx) [64,65]. This reaction can be influenced by many factors, including internal and external causes, such as the natural properties of sensitive materials, surface characteristics, the microstructure of detection layers, surface additives, UV illumination, temperature, humidity, etc. The most important performance parameters in a sensor are sensitivity; selectivity; response time; reproducibility; stability; and, of course, the limit of detection [66,67]. The articles published by Baharuddin et al. [21] and Righettoni et al. [68] show the importance of the parameters defined above considering a practical application for the detection of acetone. First of all, the sensors must show a high sensitivity toward low concentrations of acetone gas, ranging from ppt to ppm; secondly, selectivity is important because the detection of acetone in breath involves other gases or other possible breath biomarkers, especially when the diagnosis of diabetes is being considered; thirdly, the sensors should be able to operate at high relative humidity (RH) of about 90% and be resistant to its fluctuations, especially if exposed to acetone breath; and, last but not least, the response and recovery times for acetone detection are important because a “fast response” is required from gas detection devices upon being put into practice. 

### 3.2. Type of Gas Sensors 

There are several types of gas sensors, and their operation depends on the type of technology they use. Liu et al. [69] classified gas sensors into two groups according to the mode of operation: sensors that work by means of adsorption, chemical reactions, and contact with the gas and those that work based on infrared or ultrasonic emissions. Moreover, sensors (regardless of their configuration and operation) can be grouped according to the type of gas they detect. Thus, those that detect combustible gases are generally catalytic, self-powered triboelectric, and infrared sensors, while for the detection of toxic gases, the sensors generally used are electrochemical, mixed potential, and semiconductor metal oxides (SMO). 

**Figure 3:**
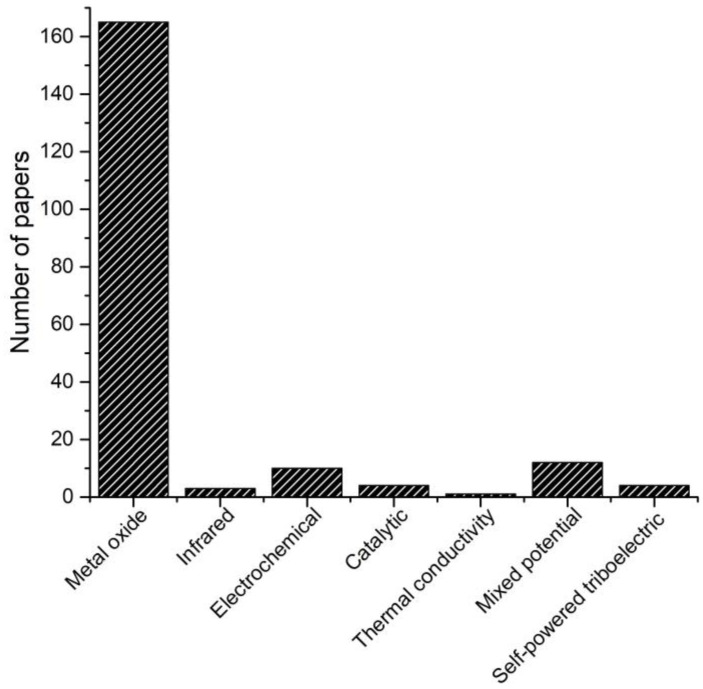
Results of the literature search (2018–2021) in the Elsevier and Scopus databases on acetone detection for different types of gas sensors.

Although there are many types of gas sensors available, in this review, the focus will be on MOx-type gas sensors due to their importance in the detection of toxic or carcinogenic gases, among which a number of VOCs have been identified. For example, acetone toxicity affects almost every system in the body, including the nervous, respiratory, cardiovascular, and endocrine systems [70]. Acetone is harmful to health, and its inhalation can cause irritation in the eyes, nose, and throat. A short, five-minute exposure at 300–500 ppm may be mildly irritating to humans. In high concentrations, it can cause dry mouth, fatigue, headache, nausea, dizziness, muscle weakness, loss of coordinated speech, and drowsiness. Ingestion can cause headaches, dizziness, and dermatitis [70]. Therefore, acetone detection encompasses a wide range of purposes—for example, the need to monitor the concentration of acetone in the environment for health and in the workplace for safety, as well as the provision of an alternative to the conventional diagnosis of diabetes. These topics have been the subject of many publications, where 83% of those are related to metal oxides (Figure 3). Therefore, in the main, only the latest results will be shown and discussed in the following sections.

### 3.3. Operation

Semiconductor devices capable of detecting the presence of certain types of gases are also of interest due to their possible miniaturization and integration, which allows for complex devices capable of detecting extremely low concentration levels to be achieved [71,72]. Currently, the most interesting semiconductors from the point of view of gas detection are certain metallic oxides: n-type semiconductors such as SnO_2_, ZnO, and TiO_2_, either in a thin or thick film form [73] or even as a ceramic body [74,75]. The choice of semiconductor as a sensor is limited to these oxides because, normally, the sensor is designed to operate in a certain atmosphere at high temperatures and in aggressive environments, and any other material tends to oxidize. Other advantages of using ceramic materials as sensors are related to the relatively simple processes of obtaining them and their low cost, which means that this type of sensor is relatively cheap [76].

**Figure 4:**
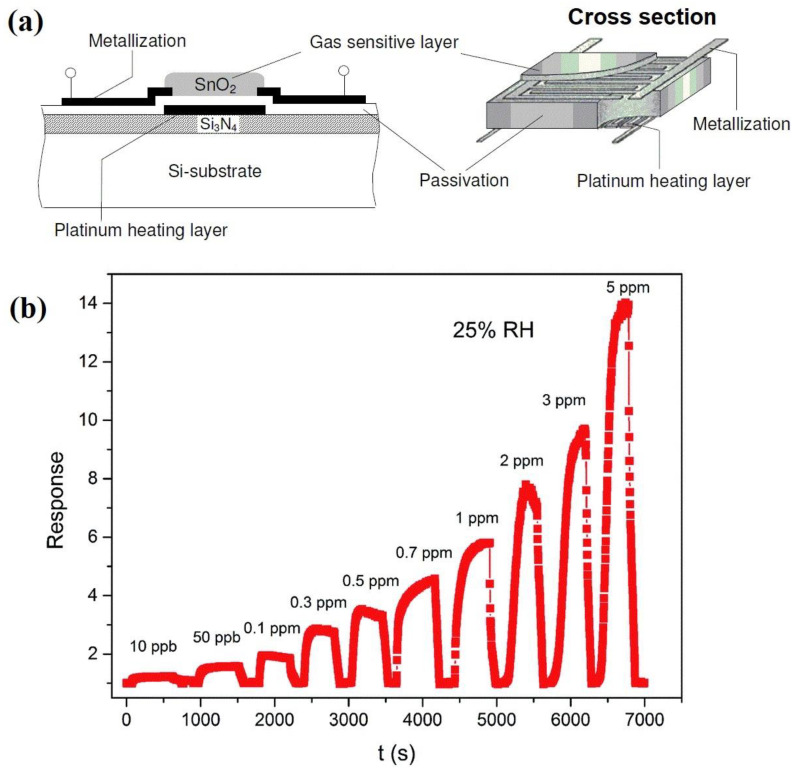
(**a**) Schematic illustration of a microstructured MOx-type gas sensor (adapted from [79]). (**b**) Characteristic curve of response and recovery toward acetone gas at different concentrations (adapted from [36]).

The operation of MOx-type gas sensors is based on the change in conductivity under the influence of reducing gases (H_2_, H_2_S, CO, NH_4_, ethanol, CH_4_, acetone) or oxidizing gases (O_2_, O_3_, NOX, CO_2_, SO_2_). Since temperature—generally referred to as the operating temperature or optimum operating temperature—is a factor that influences the behavior of the sensing material (i.e., when the sensor response is higher), these gas sensors usually include a heating element. The classical fabrication of a metal oxide gas sensor uses powder metallurgy (pellet or tube form) or thick or thin film technology [36,77]. In more advanced methods, a microstructured silicon substrate is applied as the base for the metal oxide (sensitive material) and heater. Due to the limited space to be heated and the shorter distance between the heating layer and the sensitive surface layer, the required heating power of microstructured sensors is lower than that of conventional sensors [78]. The design of a metal oxide gas sensor in microstructured silicon technology is illustrated in Figure 4a. 

Figure 4b presents the dynamic response–recovery curve as a function of the acetone concentration obtained by Chen and Cao [36] using a gas sensor based on a ZnO/SnO_2_ thick film at an operating temperature of 180 °C. The sensor signal shows an immediate response to the change in the concentration (ppm) of acetone. After several cycles between acetone gas and air, the sensor response can still recover to the initial state, indicating that the sensor has good reversibility. The sensor response was defined as R_a_/R_g_ (for a p-type semiconductor, it is its inverse: R_g_/R_a_ [80]), where R_a_ and R_g_ are the resistance in dry/humid air and the resistance after gas exposure, respectively. 

### 3.4. Mechanisms of Gas Detection

**Figure 5:**
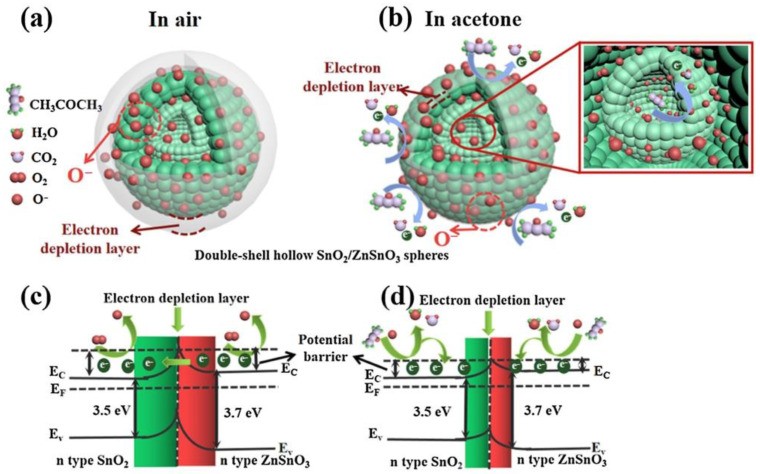
Schematic illustration of the sensing mechanism of a double-shelled hollow SnO_2_/ZnSnO_3_ composite microspheres-based sensor to acetone [40].

MOx semiconductors are characterized by their gas-sensing mechanism being controlled by the surface [66,81]. The most widely accepted model to explain sensitivity posits that changes in resistance are due to the species and amount of chemisorbed oxygen on the surface. When n-type semiconductor-based sensors are exposed to air, the electrical resistance of the material is controlled by the concentration of adsorbed oxygen species ($O_{2}^{-}$, O^−^ or O^2−^) that trap electrons (Figure 5a) and act as dispersion centers, effectively reducing their conductivity (when the working temperature is below 100 °C, the oxygen ions are mainly in the $O_{2}^{-}$ form; in the range of 100–300 °C, O^−^ ions are stable oxygen species; beyond 300 °C, the dominant oxygen species are O^2−^ ions [82]). When the sensor is exposed to acetone gas at the optimum operating temperature, the gas reacts with the adsorbed oxygen species, reducing their concentration and thus increasing the conductivity of the semiconductor, i.e., reducing the electrical resistance as observed in the measurements shown in Figure 4b. The reaction that occurs can be explained by Equation (1):

CH_3_COCH_3_ (gas) + 8O^−^ (ads) → 3CO_2_ + 3H_2_O + 8e (1)

This indicates that the acetone molecules adsorb on the surface of the sensitive material and react with oxygen ions to produce CO_2_, H_2_O, and free electrons (Figure 5b). In this process, electrons are released back into the conduction band, resulting in a substantial increase in the density of charge carriers on the surface. This reduces the width of the semiconductor depletion layer (Figure 5d) and the height of the potential barrier [82] and, consequently, the resistance of the sensitive material. This process takes place in the same way in the reaction sites of the remaining surface of the sensitive material as in the pores that are present. When the sensors are re-exposed to ambient air, the acetone gas desorbs from the surface of the material; the oxygen captures electrons from the conduction band to form oxygen ions, which increases the width of the depletion-layer electrons (Figure 5c); and the resistance of the sensitive material returns to the initial value, as seen in Figure 4b.

**Figure 6:**
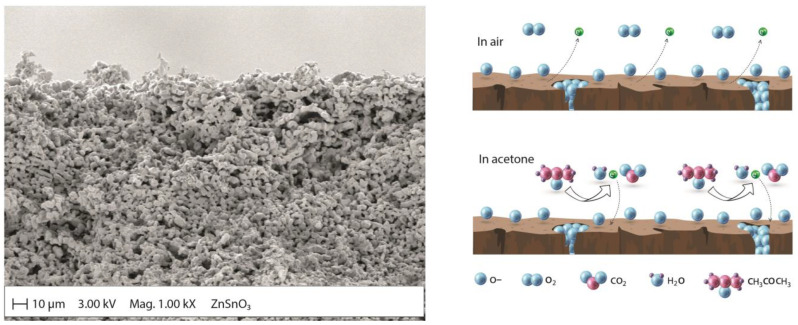
Scanning electron microscopy images and illustration of the gas-sensing mechanism of the ZnSnO_3_ sensor (adapted from [44]).

**Figure 7:**
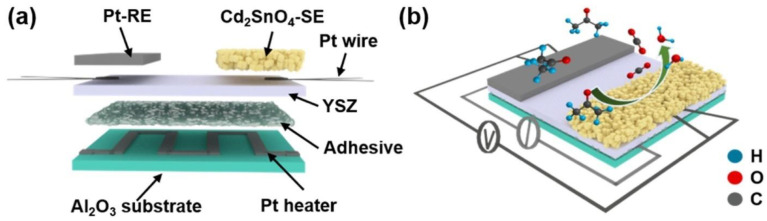
(**a**) Structure of the planar-type YSZ-based sensor and (**b**) schematic diagram of the measurement [30].

Although MOx micro/nanostructures such as SnO_2_, ZnO, and TiO_2_ have been used as gas-sensing materials for several decades due to their unique physicochemical properties, the lack of selectivity and high operating temperatures (200–600 °C) of these oxides [45] has led to the development of special MOx sensors. Improvements include doping/decoration with noble metals (for example, Pd, Ag, Pt, and Au) [83,84], surface functionalization [85,86], composite production (for example, MOx–MOx, polymer–MOx, and MOx–carbon nanotubes, among other compositions) [87]. These advances have shown that gas detection performance is primarily mediated by the surface properties of the sensing material and its combination of multiple components, which act synergistically to increase sensitivity, selectivity, and response rates during acetone detection. Chen et al. [40] obtained SnO_2_/ZnSnO_3_ composite microspheres with double-layer hollow structures using the chemical vapor deposition (CVD) method. This composite material has shown an important sensing capacity against different gases [88], among them, acetone [42], ethanol [89,90], formaldehyde [88,91], CO [92], NO_2_ [93], H_2_ [94], etc. The structure obtained by Cheng et al. [40] showed excellent selectivity and long-term stability against acetone. The authors attributed this performance to the synergistic effect of the hollow double-layer structure with a large specific surface area and the n–n heterojunction at the SnO_2_/ZnSnO_3_ compound interface. Du et al. [41] showed that multi-shelled ZnSnO_3_ hollow microspheres exhibited excellent acetone sensing performance, which was superior to that of double-shelled samples and the nanocomposites reported by other authors [40,43]. Ochoa et al. [44] formed porous bodies of MSnO_3_ (M = Ba, Ca, Zn) with slip casting. Figure 6 shows a scanning electron microscopy image and an illustration of the gas detection mechanism based on the ZnSnO_3_ porous body. The lowest measurements for acetone, ethanol, and toluene vapors in humid environments (10–30% relative humidity) were performed at 1 ppm. Hanh et al. [45] developed an acetone gas sensor using Pt–Zn_2_SnO_4_ hollow octahedra for exhaled breath analysis to diagnose diabetes. Zn_2_SnO_4_ hollow octahedra prepared by a hydrothermal method were decorated with different amounts of Pt nanoparticles through a simple mixing process to optimize the Pt content to enhance the acetone-sensing performance. Liu et al. [37] designed a room temperature-operating triboelectric acetone sensor (TAS) based on a chitosan (CTS)/zinc oxide (ZnO) bilayer film (CTS/ZnO-TAS) with good humidity-tolerant properties (89.3% RH), demonstrating a potential application in noninvasive diabetes diagnoses. Jiang et al. [30] fabricated a zirconia (YSZ)-based mixed-potential-type acetone sensor using a Cd_2_SnO_4_ sensing electrode. The developed sensor detected acetone in a concentration range of 0.05–200 ppm at an optimal operating temperature of 600 °C. The sensing mechanism was analyzed and verified through the test of polarization curves (Figure 7). In addition, they carried out clinical tests for the sensor on the exhaled breath of healthy people and diabetic patients, and strong correlations were established between the response values, acetone concentrations, and blood ketone concentrations, which demonstrate the great potential of the sensor for the prediagnosis of diabetes.

Different materials, structures, and morphologies for gas-sensing layers could further enhance the performance of nanosensors and allow for the suitable detection of biomarkers even with highly humidified samples and at high levels of interfering gases due to existing cross-sensitivity. Therefore, breath analyzers with excellent selectivity and good moisture-resistant properties still need to be intensively investigated, which is one of the biggest challenges in realizing online breath analyses. These requirements are the motivation for all researchers to develop a fast, cheap, and highly sensitive biomarker detector. In the following section, the latest achievements of selected MOx sensors in acetone measurement are presented.

## 4. Results of Acetone Detection in MOx Sensors

### 4.1. ZnO-Based Sensors

Zinc oxide (ZnO) is an n-type semiconductor metal oxide. The first report of its use as a gas sensor dates to 1962 [95]. ZnO has been extensively researched as a base material for acetone detection due to its high electron mobility, chemical stability, and structural adjustability [35,68]. ZnO (as well as SnO_2_) is one of the few materials to have been successfully commercialized. However, there are still some drawbacks, such as high working temperatures, low sensitivity, and poor selectivity, which make it difficult to apply in practice as a high-performance gas sensor. Van Duy et al. [34] studied the performance of acetone detection using ultrafine porous ZnO nanoplates obtained with the hydrothermal method, showing responses of 20 at an exposure of 125 ppm evaluated at 450 °C, a relatively high operating temperature compared with other reports of similar morphology [96]. However, new compositions of ZnO nanostructures in synergy with other components continue to be researched in terms of improving the detection of acetone at lower concentrations. Recently, Wang et al. [35] used Au-functionalized ZnO flowers with thin film and a hemispherical morphology, obtained via thermal decomposition and with responses of 2900 measured at 100 ppm acetone vapor operating at 365 °C. Drmosh et al. [38] fabricated Au-decorated ZnO/Ag core–shell films via sequential DC sputtering for the detection of acetone in low concentrations by engineering the depletion layer. The acetone-sensing performance of Au/ZnO/Ag is four- and two-times, respectively, higher than that of ZnO and ZnO/Ag core–shell films. Chen and Cao [36] synthesized ZnO/SnO_2_ hybrid nanospheres using the sol–gel method; the authors formed thick films with improved detection properties at a relatively low operating temperature (180 °C) and a low detection limit (ppb level) for the detection of acetone, as seen in Figure 3a. The reported sensitivities at 0.01 and 5 ppm acetone were 1.23 and 13.83, respectively. These results are considered promising when the sensor is required to correlate the detection of low concentrations of acetone with diabetes. 

### 4.2. SnO_2_-Based Sensors

Among the various semiconducting metal oxides, tin oxides are the most popular gas detection material researched so far and used in practice, including for the improved detection of acetone [72]. Recently, Hu et al. [39] fabricated structures of NiO/SnO_2_ (p/n) compounds using the hydrothermal method and evaluated the detection of acetone-type gases in a temperature range of 200 to 400 °C; the maximum response was 20.18 measured at 300 °C up to 50 ppm acetone [58]. Kalidoss et al. [48] investigated using ternary nanocomposite graphene oxide–tin dioxide–titanium dioxide (GO-SnO_2_-TiO_2_) to detect acetone in the breath of patients with diabetes. These sensors exhibited superior performance in a range of 0.25 to 30 ppm at 200 °C; at the 3 ppm exposure, the response was ~13. The ternary nanocomposite sensor developed by Kalidoss et al. [48] had short response and recovery times—10 s and 12 s, respectively—measured at 3 ppm acetone vapors.

### 4.3. TiO_2_-Based Sensors

Titanium oxide (TiO_2_), a typical n-type semiconductor and has been featured in research and industry due to its excellent physicochemical properties, low cost, and simple synthesis method [97]. In particular, TiO_2_-based gas sensors exhibit not only good stability but also fast response/recovery times [49]. However, most pure TiO_2_ sensors have shown a poor response to VOCs, especially acetone. Navale et al. [49] synthesized TiO_2_ particles using the hydrothermal method. The response was only four to 100 ppm acetone at an optimal operating temperature of 270 °C. Chen et al. [98] used TiO_2_ nanospheres prepared with the sol–gel method. The response at 1000 ppm acetone reached only 2.3. Bhowmik et al. [99] reported that the response of TiO_2_ nanoflowers, obtained using the hydrothermal process, was even lower than three at 700 ppm acetone. From the point of view of practical applications, the above works still cannot meet the requirements for the high-performance detection of acetone. Different strategies have been proposed to improve the gas detection performance of TiO_2_-based sensors, such as the construction of heterojunctions between different semiconductor oxides [100]. Y. Zhou et al. [50] prepared a TiO_2_/Ag_2_V_4_O_11_ nanostructure gas sensor, and its response at 100 ppm acetone was 10.2, a higher response than pure TiO_2_ (3.4). Lou et al. [101] synthesized nanofibers composed of TiO_2_-Fe_2_O_3_ and the response to 50 ppm acetone was about three times higher than that of pure TiO_2_ and Fe_2_O_3_. Sharma et al. [51] reported thin films based on TiO_2_-SnO_2_ heterostructures obtained with the PVD method and exposed to different concentrations of acetone (0.25 to 100 ppm) at a temperature of 300 °C. The sensors proposed by Sharma et al. [51] showed high responses, selectivity, and long-term stability (measured up to 60 days). The design and synthesis of heterostructures with controllable morphologies and compositions in the fabrication of TiO_2_-based gas sensors can, therefore, lead to high-performance sensors for gas detection. 

### 4.4. WO_3_-Based Sensors

One of the metal oxides commonly used for the detection of exhaled acetone is tungsten oxide (WO_3_), which has n-type conductive behavior with a catalytic effect both in the oxidation and reduction reactions on its surface. Recently, Muñoz and Rodríguez [52] used colloidal processing (slip casting) to control the elaboration of sensitive materials, that is, macroporous bodies based on WO_3_. These pieces showed good sensitivity to acetone vapors at concentrations above 1 ppm and at 250 and 300 °C. Zhang et al. [53] proposed acetone sensors based on WO_3_ compounds modified with Au, with macroporous architecture and a detection limit of 100 ppb at 410 °C, a sufficient value suited to analyzing breath acetone for diabetes screening. The W_18_O_49_:Pt studied by Xu et al. [54] demonstrated excellent sensing performance with a response of 85 upon exposure to 20 ppm of acetone at an operating temperature of 180 °C. Lu et al. [55] presented the detection results of WO_3_ nanocrystals obtained with the hydrothermal method. WO_3_ sensors were studied in an acetone vapor concentration range of 0.25 ppm–100 ppm, yielding responses of 3.8–250 ppb and 31–100 ppm, respectively, at an optimum operating temperature of 320 °C. The detection limit was estimated as 7.5 ppb, which is applicable for the detection of ultra-low concentrations, for example, in the noninvasive diagnosis of diabetes.

### 4.5. FexOy-Based Sensors

The most common iron oxides are FeO, Fe_2_O_3_, and Fe_3_O_4_, with Fe_2_O_3_ generally being the most widely studied in gas detection applications. The main limitation for Fe_2_O_3_-based gas sensors is the operating temperature (450–1075 °C), as the operating temperature is difficult to implement on gas sensor substrates such as silicon. However, recently, Zahmouli et al. [56] obtained samples synthesized using the sol–gel technique on pure γ-Fe_2_O_3_ doped with different loads of gadolinium. These doped sensors exhibited sensitivities to low acetone concentrations (~1 ppm) and lower temperatures (200 °C). The best detection performance was with a 3% Gd load, with a response (R_g_/R_a_) of 31.23 against 20 ppm acetone, 30 times higher than that of pure γ-Fe_2_O_3_. This novel sensor showed good selectivity and signal stability/response reproducibility, which makes it very promising for practical applications, and it can very likely attract more attention in the future. 

### 4.6. In_2_O_3_-Based Sensors

Cubic indium oxide (In_2_O_3_) has been widely used in the microelectronic field, including in gas sensors, due to its lack of stoichiometry. Nonstoichiometric defects arise due to the presence of an excess or deficiency of metal ions, which, in turn, is highly dependent on the synthesis process. These defects induce modifications in the band structure of the ideal In_2_O_3_, thus varying its electrical resistance [102,103]. Che et al. [62] presented results related to In_2_O_3_ microstructures with nanowire morphologies, fabricated by electrospinning exposed to acetone environments. The maximum response obtained to 100 ppm of acetone was 37.9 at an optimum operating temperature of 200 °C. The developed sensor was able to identify a variety of VOC gases, reflecting good selectivity; it also had a very fast response/recovery time (1/7 s) and long-term stability characteristics (measured up to 30 days) against acetone gas. However, there remains the need to determine its sensitivity to low concentrations of acetone in humid environments, conditions similar to exhaled breath. 

### 4.7. CuO-Based Sensors

In most of its compounds, copper has low oxidation states, the most common being +2, although there are also some with oxidation states of +1. The CuO phase, however, is considered a gas-sensitive material with p-type semiconductor properties. Wang et al. [58], using sensors based on CuO powders, reported responses of only ~2 to 500 ppm acetone operating at room temperature; even when using Cu_2_O–CuO porous octahedrons, no sensitivity enhancement to these vapors was achieved. Previous studies developed by Choi et al. [59]—using Cu_2_O–CuO composites decorated with Ag nanoparticles evaluated at different concentrations of acetone (0.125 to 1000 ppm) and an operating temperature of 350 °C—reported superior results to undecorated structures (8.0 to 0.125 ppb and 34 at 1000 ppm) with the following recovery times: 27 s for 125 ppb and 37.9 s for 1000 ppm acetone. The sensor developed by Choi et al. [59], on the one hand, exhibited good sensitivity to acetone at lower concentrations, but, on the other hand, the operating temperature was very high compared with the latest achievement in the field.

### 4.8. Light-Assisted Detection

**Figure 8:**
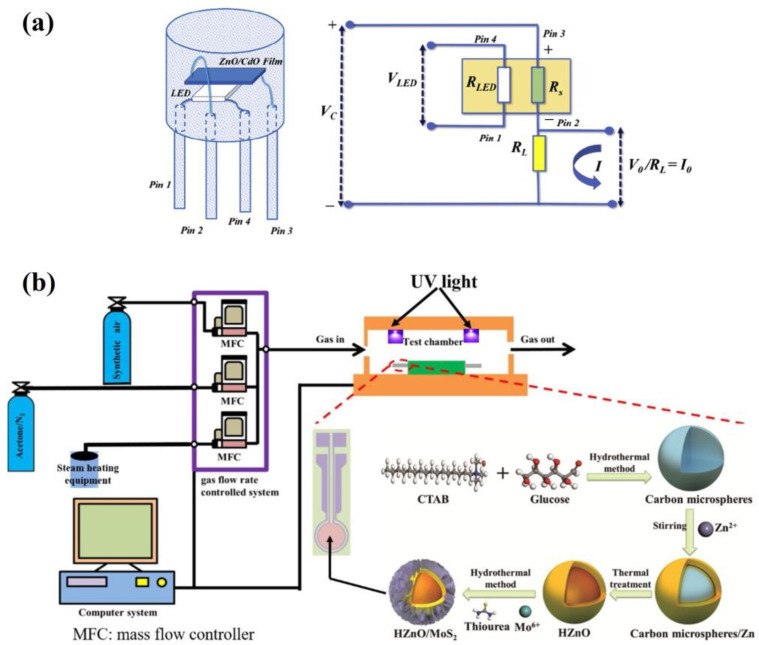
Diagram of (**a**) ZnO/CdO heterostructure sensor device and equivalent circuit (adapted from [60]); (**b**) gas-sensing test system and fabrication processes of HZnO/MoS_2_ core–shell heterogeneous structures (adapted from [61]).

Apart from thermal excitation, one of the promising methods of increasing the limit of detection for metal oxide sensors is ultraviolet (UV) illumination during gas detection. Photoexcitation induces electron–hole pairs [61,104], a phenomenon that increases the width of the accumulation layer and reduces the width of the depletion layer, thereby promoting the adsorption and desorption processes of the surface oxygen molecules, achieving a rapid response and recovery against acetone vapors at low temperatures. Recent studies have shown detection tests with UV illumination on heterostructures based on ZnO due to the ease of UV activation shown by this semiconductor metal oxide [23]. In 2019, Srinivasan et al. [60] studied the effect of UV irradiation (UV LED light) on the response of ZnO/CdO heterostructure thin film obtained with the spray pyrolysis technique. The sensitive film showed a selective photoresponse of 540 to 1 ppm acetone at room temperature, and the response and recovery times were 61 and 47 s, respectively. The authors found that UV irradiation reduced surface band bending, which promoted interparticle charge transfer and reduced sensor baseline resistance drift. It also stimulated the desorption of analytes, increasing the reproducibility of sensor signals. The configuration of the sensor device and its equivalent circuit is shown in Figure 8a. UV LED (blue light) with a wavelength of 365 nm was employed as a light source in which the V_LED_ was chosen as 2.1 V to ensure an irradiated power density of 3.01 mW/cm^2^. In 2020, Chang et al. [61] examined the acetone-sensing properties of hollow ZnO/MoS_2_ nanosheets under UV illumination. Core–shell heterostructures were synthesized through a simple hydrothermal method. Light-emitting diode-based UV light (375 nm, 50 μW/cm^2^) was applied to assist the acetone-sensing measurement, as shown in Figure 8b. In this study, it was reported that the acetone-sensing properties obtained by the synergistic effect of the UV light and HZnO/MoS_2_ core–shell heterogeneous structures showed a stable response (1.52) to 100 °C and 100 ppb acetone with UV irradiation while exhibiting no response to 100 ppb acetone without UV. Even at room temperature (30 °C), UV-activated HZnO/MoS_2_ still exhibited a stable response (∼1.33) and fast recovery time (19 s/97 s) to 50 ppm acetone. Among other photo-assisted structures are the sensors developed by Wang et al. [62], Fe-doped hexagonal and monoclinic WO_3_ synthesized using the solvothermal calcination method. Under LED illumination and an operating temperature of 260 °C, the optimized 1.25Fe–h/m–WO_3_ sensor exhibited higher responses to acetone compared with other identically prepared sensors, and excellent linearity between responses and acetone concentration (0.5–2.5 ppm) was achieved at 90% RH. Meanwhile, the 1.25Fe–h/m–WO_3_ sensor exhibited good acetone selectivity and stability over three months. In this work, photocatalytic performance in the degradation of rhodamine B (RhB) was also studied under visible-light illumination. 

Guo et al. [63] developed an acetone gas sensor based on a CdS/Co_3_O_4_ nanocomposite using an electrospinning method combined with a hydrothermal method. They used green visible light to improve the sensing properties of the CdS/Co_3_O_4_ sensor operated at room temperature (25 °C). With the irradiation of 520 nm green visible light, the response of CdS/Co_3_O_4_ to 50 ppm acetone increased by about 25%, and the response/recovery time was shortened to 5 s/4 s. The excellent sensing performance of the nanocomposite was mainly attributed to the one-dimensional-structured morphology and the formation of p-n heterojunctions, as well as the catalytic activity of Co_3_O_4_ on acetone gas. Since the revision of the sensors under photoexcitation showed the lowest detection limits of 1 ppm, these can be used for the identification of acetone from exhaled breath for the diagnosis of diseases. 

### 4.9. Comparison

In recent decades, MOx-based gas sensors have been used primarily to detect VOCs (sub-ppm) in human breath due to their low cost, compact size, ease of production, and simple measuring electronics. These materials include TiO_2_, WO_3_, ZnO, Fe_2_O_3_, In_2_O_3_, CuO, and SnO_2_, to name just a few. Specifically, ZnO and WO_3_ are sought after as sensors for their high sensitivity to VOCs such as acetone, in addition to their ability to present various crystalline and complex nanoparticle morphologies, as discussed in the work of Jing et al. [105] and Lu et al. [55]. In addition, In_2_O_3_ has shown very promising results in the detection of acetone [57]. The current review includes single-oxides, but most of the investigated gas-sensitive materials are doped with other metals, such as Au, Ag, Pt, Gd, and Fe, or correspond to multi-oxide systems with the hope of improving their performance. However, the versatility of these metal oxides comes at a price. Historically, simple metal oxides had poor stability with cyclical exposure to moisture and various test gases. Progress has been made in improving stability, but there is a limit to this because the sensor will always be in direct contact with test gases and moisture. Light-assisted sensors can be beneficial in these situations; they use a closed-loop system to protect the sensors and offer long-term stability, as per Wang et al. [62].

The most prominent sensors discussed here are semiconductor metal oxides. They differ in their composition, operating temperature, output data (selectivity, sensitivity, response), and the breadth of their tests. Moisture testing is necessary because human breath is very humid, and before human testing can begin, the sensor must be validated under these conditions. Many sensors have been tested in moist conditions and even tested for long-term stability in moist conditions [30,40,62], which is very valuable. More than half of the sensors discussed here have not been tested on humans. In the early stages of development, this is acceptable, but to be considered for use as commercial devices, they must undergo clinical validation.

The selectivity of the sensors is very important to differentiate between different VOCs. The highest selectivity was observed in several works [35,42,51,53], highlighting the study by Xu et al. [54]. This is due to the control they carried out to manufacture the sensing layer. They also determined one of the lowest LODs (0.0052 ppm) of the MOx discussed in this article. The maximum response for each sensor was also noted, and the highest was noted with the sensor of Wang et al. [35], with a response of 2900 (R_air_/R_gas_) at 100 ppm, without considering the effect of humidity. The maximum response is important because it must be significant enough to be detected, but it must also vary from other test gases to have good selectivity. Response and recovery times are also crucial because they allow for comfort and ease of use. A sensor with a long response or recovery time may not be commercially successful. The lowest times reported were with the mixed potential sensor from Jiang et al. [30] with 2/8 s response and recovery times, respectively, at 10 ppm acetone and an operating temperature of 600 °C. The operating temperature is another crucial parameter. A high operating temperature can be dangerous and causes sensitive material to deteriorate. A lower temperature is preferred to keep the sensor stable [19]. The light-assisted sensors all detected acetone vapors at room temperature.

For a manufacturer to decide on the use of different technologies, it all comes down to its needs. A high operating temperature sensor may be suitable if it is inexpensive, and the company can afford the excess power needed for a new power supply system. A sensor with low selectivity may be successful with a company that then adds an earlier step to reduce other analytes, such as the design of Rydosz et al. [106], with a preconcentrator chip. There is no sensor that is the best in all respects, but there are certainly sensors that have valuable features.

## 5. Devices and Applications in Breath Gas Analysis

**Figure 9:**
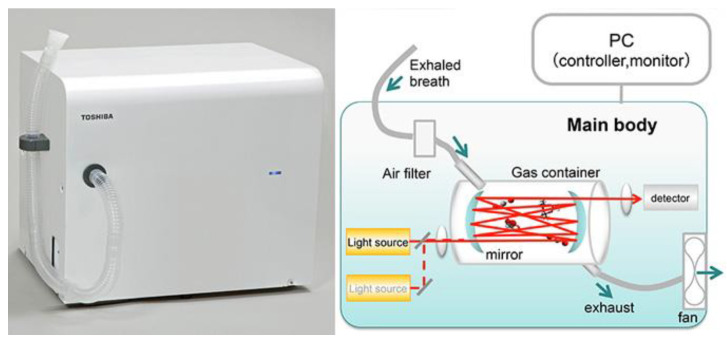
Toshiba developed a breathalyzer to determine health problems [107].

**Figure 10:**
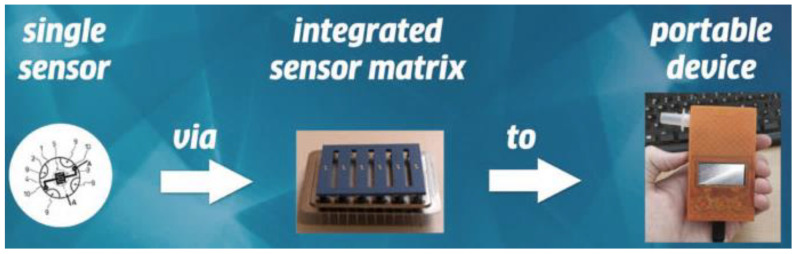
“Diabetomat” developed by Dr. Artur Rydosz (adapted from [108]).

**Figure 11:**
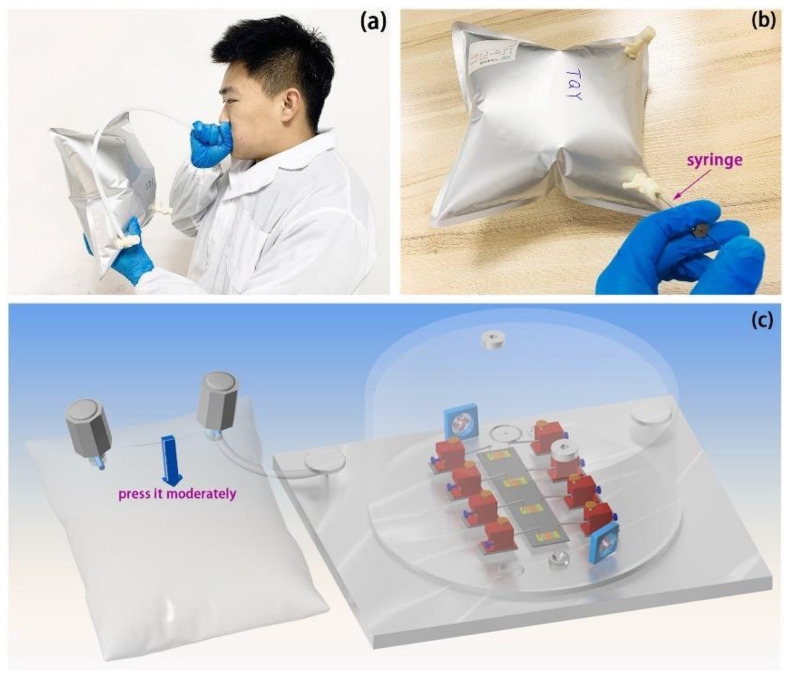
(**a**,**b**) The process of obtaining the simulated exhaled gas of diabetic patients. (**c**) A schematic diagram depicts how the simulated exhaled gas of diabetic patients is tested [46].

**Figure 12:**
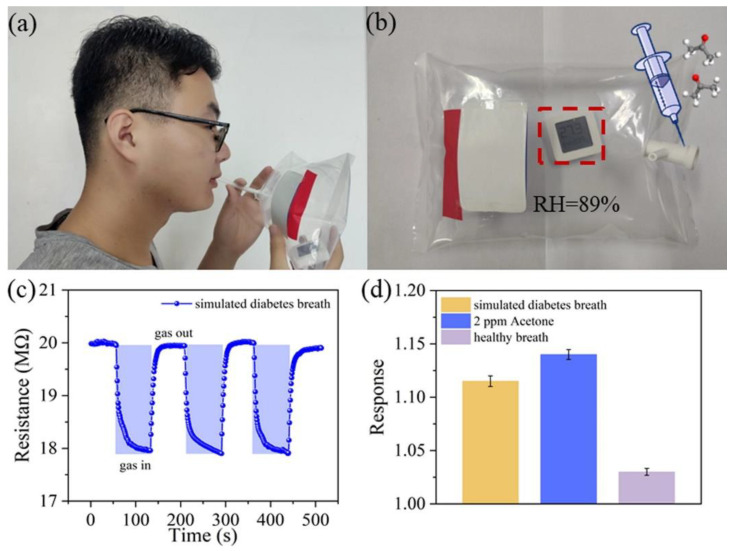
Investigation of the breath gas analysis of a Co_3_O_4_/CdS sensor. (**a**) The breath of a healthy experimenter is collected with an air bag. (**b**) The breathing of diabetic patients is simulated by injecting 2 ppm acetone into the exhaled breath of healthy people. (**c**) The response curve of the sensor in the environment of the simulated breath of diabetic patients. (**d**) The response value of the sensor tested upon exposure to simulated diabetes breath, 2 ppm acetone, and healthy breath [63].

There are several potential advantages to breath tests over conventional laboratory tests (for example, noninvasive, painless, easy-to-use, real-time measurements, etc.). However, they have not yet been applied to clinical practice, possibly because there are no commercially available devices. In 2014, Toshiba Corp. [107] announced that they had developed a prototype of a compact breath analyzer that can detect a wide range of target gases in exhaled breath (Figure 9). The current version of the equipment can detect acetaldehyde, methane, and acetone. By analyzing these trace gases, it is possible to measure one’s health and metabolic activity. That analysis can be performed by simply having the subject breathe and is less stressful on the body and mind than blood tests and urinalysis. In 2020, Dr. Artur Rydosz created the “Diabetomat” (Figure 10) [108]. The device analyzes the composition of exhaled air, measures the content of ketone bodies, and relates this to the blood glucose level. Additionally, this system uses the pre-concentration technique to reduce the moisture level in the exhaled samples [106] (integrated sensor matrix in Figure 10). In 2022, an ultrasensitive acetone gas sensor based on a p-Rh_2_O_3_-n-ZnO porous heterostructure was fabricated by Cai et al. [46]. Figure 11 shows the process of obtaining the simulated exhaled gas of diabetic patients. A volume of 5 L exhaled gas from the healthy people was obtained. Acetone was then injected into the bag to add a concentration of 900 ppb acetone into it, raising the acetone concentration in the bag to 1200–1800 ppb since 300–900 ppb and 1800 ppb acetone was contained in the exhaled gas of the healthy people and the diabetic patients, respectively. Guo et al. [63] investigated the feasibility of CdS/Co_3_O_4_ composite material for breath analysis. They used a bag to collect the exhaled air of healthy people and mixed this with 2 ppm of acetone gas to simulate the breathing environment of a diabetic patient (Figure 12a,b). The hygrometer in the airbag showed that the humidity of the exhaled air was 89%, and the humidity of the test environment was controlled at this level to avoid the influence of humidity. Figure 12c,d show the repeatability of the sensor in the environment of the simulated breath of diabetic patients and healthy breath. Most of the devices developed are still under laboratory verification or in clinical trials, studies that are crucial before market entry. 

## 6. Conclusions and Futures Prospects

In this review, metal oxide (MOx) gas sensors were investigated. A comparative study was presented of different micro- and nanostructures for the detection of acetone considering their potential use in the diagnosis of diabetes. The general properties and their gas detection mechanisms, with porous architecture, were also considered. This effort was aimed at providing researchers and industrialists working in the field of VOCs, especially in the detection of acetone, with valuable data and benchmarks. The advances described here suggest that the MOx semiconductor nanomaterial-based acetone detection technique is rapidly thriving. Therefore, a comprehensive understanding of recent acetone gas sensor development is essential to ensure rapid progression is maintained in the future. 

As illustrated in the course of this review, several sensors have been developed using different metal oxide-based nanomaterials, synthesis methods, and sensor modalities. The procedures and protocols to evaluate the multiple sensors vary widely from one publication to another, since there is no norm or standard that provides the rules to execute them, and, therefore, cross-comparisons are a challenge. However, the most commonly studied reliability parameters for acetone detection are shown, including sensitivity, selectivity, response/recovery time, operating temperature, stability, and some significant sensor parameters for the selected functions. Promisingly, these analyzed parameters are improved in gas sensors by doping/decorating them with noble metals, surface functionalization, and the formation of MOx heterostructures. The heterojunctions allow for effective conduction channel tuning, and the synergistic effect of various semiconductors with respect to their gas detection performance. The main drawbacks of the reviewed nanocomposites are their higher operating temperatures. This can be reduced by working on the concentration of the component materials, as well as controlling the morphology, although this parameter was not a central idea in this review. It should also be noted that thermal and optical excitations with UV increase surface defects and, therefore, the effective enhancement in the charge carriers for the more efficient adsorption and diffusion of gases is a facet of gas-sensing which are still under improvement. However, ultraviolet light consumes a lot of light and has radiation. Compared with ultraviolet light, visible light is cheap and environmentally friendly, but the excitation rate is low. Hence, in subsequent research, the optical properties of the material itself should be adjusted to achieve a high excitation rate and enhance gas-sensing performance under visible light irradiation. This, therefore, offers a gap for future researchers in gas-sensing applications.

Efforts are currently being made in the medical and engineering sectors to manufacture acetone sensors that incorporate improved sensitivity, capable of detecting low concentrations of biomarkers, such as acetone, and also require less thermal energy, among other characteristics of the sensor. The development of the breath acetone sensor in the coming years ought to focus on the manufacturing process in order to deliver to the market a final device for accurately and reproducibly collecting exhaled breath and performing an analysis thereof. Its instrumentation ought to consider variations in the respiration cycle (for example, the way human subjects breathe during measurements) and the background level of interfering compounds (for example, ambient air pollution is an issue). Long-term tests must be carried out for all the aforementioned detection techniques in order to be one hundred percent sure that the results obtained are relevant. Multidisciplinary collaboration is the sole way to achieve that goal: the development of totally noninvasive devices for the detection and evaluation of states of disease. 

## Figures and Tables

**Table 1:** Summary of the literature review of selected metal oxide-based sensors for acetone detection published between 2019 and 2022.

Sensitive Material	^1^ Response	^2^ T (°C)	C_(acetone)_ (ppm)	^3^ LOD (ppm)	Reference
YSZ-Cd_2_SnO_4_	* 60–70	600	10/98% RH	0.05	[30]
ZnO	~12	450	125/80% RH	~2	[34]
ZnO: Au	2900	365	100	–	[35]
ZnO/SnO_2_	13.83	180	5/25% RH	0.5	[36]
Chitosan/ZnO	~18	RT	10	1	[37]
Ag/ZnO/Au	** 80	150	5	1	[38]
NiO/SnO_2_	20.18	300	50	0.01	[39]
SnO_2_/ZnSnO_3_	16.7	290	100/90% RH	2	[40]
ZnSnO_3_	105.1	260	100/65% RH	–	[41]
ZnSnO_3_/SnO_2_	19.1	260	50	1	[42]
ZnFe_2_O_4_/ZnSnO_3_	63.3	200	30/50% RH	–	[43]
ZnSnO_3_ bodies	37	270	80/30% RH	1	[44]
Zn_2_SnO_4_:Pt	33	300	100	1.27	[45]
Rh_2_O_3_-ZnO	1.9	250	0.0100	0.005	[46]
RGO	31.23	RT	1000	–	[47]
GO-SnO_2_-TiO_2_	60	200	5	0.25	[48]
TiO_2_	15.24	270	1000	0.5	[49]
TiO_2_/Ag_2_V_4_O_11_	10.2	300	100/30% RH	–	[50]
TiO_2_/SnO_2_	303.5	300	100	0.02	[51]
WO_3_	–	250	>1	–	[52]
WO_3_:Au	7.6	410	1.5	0.1	[53]
W_18_O_49_:Pt	~6	180	20/95%	0.0052	[54]
WO_3_	3.8	320	0.25	0.0075	[55]
γ-Fe_2_O_3_:Gd	31.2	200	20	–	[56]
In_2_O_3_	39.7	200	100	–	[57]
In_2_O_3_/TiO_2_	33.34	250	10	0.1	[27]
Cu_2_O-CuO	25	RT	500/30% RH	–	[58]
Cu_2_O-CuO:Ag	** 34	350	1000	–	[59]
ZnO/CdO	*** 540	RT	1	1	[60]
ZnO/MoS_2_	*** 1.33	RT	0.001	0.001	[61]
WO_3_:Fe	*** 12	260	10/90% RH	0.2	[62]
CdS/Co_3_O_4_	*** 7.22	RT	100	0.5	[63]

^1^ R_a_/R_g_: maximum electrical resistance under exposure to air/humid and target gas (acetone), respectively, for n-type semiconductors; R_g_/R_a_: for p-type semiconductors. ^2^ T(°C): Operating temperature. ^3^ LOD: Limit of detection. RT: Room temperature. * ΔV (mV). ** ΔR/R_a_ (%). *** In the presence of light.
